# A Quantitative Trait Locus with a Major Effect on Root-Lesion Nematode Resistance in Barley

**DOI:** 10.3390/plants13121663

**Published:** 2024-06-15

**Authors:** Diane Mather, Elysia Vassos, Jason Sheedy, Wenbin Guo, Alan McKay

**Affiliations:** 1 School of Agriculture, Food and Wine, Waite Research Institute, The University of Adelaide, Adelaide, SA 5005, Australia; elysia.vassos@grainsaustralia.com.au; 2 Centre for Crop Health, University of Southern Queensland, Toowoomba, QLD 4350, Australia; jason.sheedy@usq.edu.au; 3 Information and Computational Sciences, The James Hutton Institute, Dundee DD2 5DA, UK; wenbin.guo@hutton.ac.uk; 4 South Australian Research and Development Institute, Adelaide, SA 5001, Australia; alan.mckay@sa.gov.au

**Keywords:** barley, root-lesion nematode, resistance, quantitative trait locus, chromosome 7H

## Abstract

Although the root-lesion nematode *Pratylenchus thornei* is known to affect barley (*Hordeum vulgare* L.), there have been no reports on the genetic control of *P. thornei* resistance in barley. In this research, *P. thornei* resistance was assessed for a panel of 46 barley mapping parents and for two mapping populations (Arapiles/Franklin and Denar/Baudin). With both populations, a highly significant quantitative trait locus (QTL) was mapped at the same position on the long arm of chromosome 7H. Single-nucleotide polymorphisms (SNPs) in this region were anchored to an RGT Planet pan-genome assembly and assayed on the mapping parents and other barley varieties. The results indicate that Arapiles, Denar, RGT Planet and several other varieties likely have the same resistance gene on chromosome 7H. Marker assays reported here could be used to select for *P. thornei* resistance in barley breeding. Analysis of existing barley pan-genomic and pan-transcriptomic data provided a list of candidate genes along with information on the expression and differential expression of some of those genes in barley root tissue. Further research is required to identify a specific barley gene that affects root-lesion nematode resistance.

## 1. Introduction

Root-lesion nematodes (*Pratylenchus* spp.) are polycyclic migratory endoparasites of plant roots. They enter roots from the soil and migrate through root tissues. They can exit from and re-enter roots and deposit eggs both within and outside of roots. They cause damage by feeding on plant cells, mostly within the root cortex. They have broad host ranges [1].

In Australia, *Pratylenchus thornei* causes serious damage to bread wheat (*Triticum aestivum* L.) [2] and chickpea (*Cicer arietinum* L.) [3], particularly in dry conditions and on poor soils [1]. The problem is exacerbated when susceptible host crops are grown consecutively because the number of *P. thornei* in the soil rise, increasing the inoculum load for the following season’s crop. Genetic variation for *P. thornei* resistance (ability to suppress nematode multiplication) has been reported for bread wheat [4,5,6,7] and some related species [8,9,10] and for chickpea [11] and some related species [12]. Quantitative trait loci (QTLs) for *P. thornei* resistance have been mapped in bread wheat [13,14,15,16,17,18] and in chickpea [19].

*Pratylenchus thornei* can also infect barley (*Hordeum vulgare* L.). Although barley is generally considered to have moderate resistance [20], grain yield is affected, with losses estimated to cost the Australian barley industry AUD 5 million per year [21]. Resistance ratings of current cultivars in Australia range from MS (moderately susceptible) to RMR (resistant—moderately resistant) [22]. Quantitative trait loci for resistance against some other root-lesion nematodes (*P. neglectus* and *P. penetrans*) have been mapped in barley [23,24], but there are no published reports on the genetic control of *P. thornei* resistance in barley. 

The aim of this research was to investigate whether differences in *P. thornei* resistance in barley could be attributed to one or more QTLs and to develop molecular markers for use in barley breeding. The approach taken involved (1) evaluation of *P. thornei* resistance in a panel of barley mapping parents; (2) selection of mapping populations based on the panel results; (3) evaluation of *P. thornei* resistance in the selected mapping populations; (4) QTL analysis using existing linkage maps for the populations; (5) improvement of linkage maps of a chromosome on which a highly significant QTL was detected; (6) anchoring of a QTL region to a genome assembly; and (7) investigation of existing transcriptomic data for predicted genes within a candidate region for the QTL. 

## 2. Results

### 2.1. Variation for Pratylenchus thornei Resistance in Barley

A panel of 46 barley lines was evaluated for *P. thornei* resistance using inoculated plants grown for eight weeks in a glasshouse in South Australia. The panel included 32 varieties (24 from Australia, 2 from Canada, 2 from Japan and 1 from each of Germany, the Czech Republic, France and Mexico), 12 breeding lines (4 from Australia, 4 from the USA and 1 from each of Brazil, Canada, Mexico and Uruguay), a selection from a North African landrace and an accession of wild barley (*Hordeum vulgare* ssp. *spontaneum*) (Table S1). These lines were chosen because each of them had previously been used as a parent to develop one or more populations for genetic mapping. Best linear unbiased estimates (BLUEs) for the final number of *P. thornei* nematodes per g of soil and roots (as estimated using a DNA-based method [25,26]) ranged from 4.0 to 13.0 (Table S1). A similar range was observed for eight previously well-characterized wheat (*Triticum* spp.) varieties that were included as controls (Table S1). 

### 2.2. Genetic Mapping: Arapiles/Franklin

**Figure 1:**
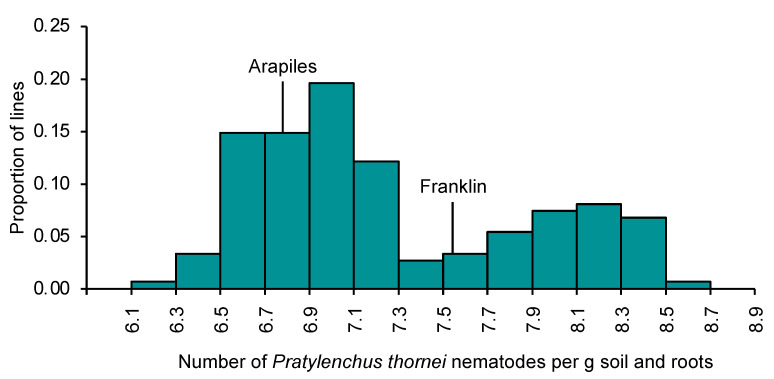
Frequency distribution for estimated final numbers of *Pratylenchus thornei* nematodes at eight weeks after inoculation for an Arapiles/Franklin barley population.

The Arapiles/Franklin population was chosen for further investigation because Arapiles (4.04 *P. thornei* per g of soil and roots) was more resistant than Franklin (10.50 *P. thornei* per g of soil and roots). Arapiles/Franklin doubled haploid lines were evaluated for *P. thornei* resistance using the same methods that had been used for the panel of mapping parents. The phenotypic frequency distribution deviated significantly from normality (Shapiro–Wilk *W =* 0.92, *p* < 0.0001) and was bimodal (Figure 1), which could indicate segregation of a major-effect locus. 

**Figure 2:**
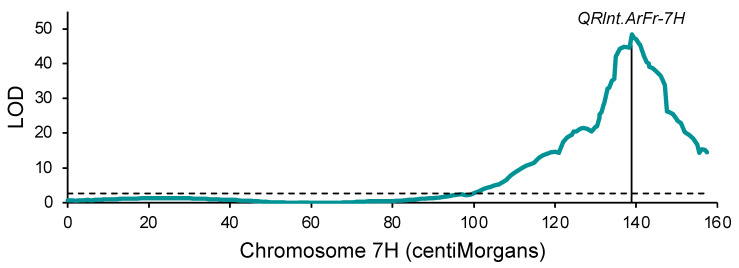
Quantitative trait locus *Rlnt.ArFr-7H* for *Pratylenchus thornei* resistance mapped in an Arapiles/Franklin barley population using a linkage map of single-nucleotide polymorphisms on chromosome 7H. The vertical line shows the position of marker *wri907*. The horizontal dashed line shows the genome-wide significance threshold (LOD = 2.69).

With QTL analysis using an existing molecular marker map (Table S2) that had been developed to map QTLs for malting quality traits [27], a major QTL (LOD = 36.3) was mapped at 222.0 centiMorgans (cM) on chromosome 7H (Figure S1). At this QTL, the favorable effect (fewer *P. thornei*) was from Arapiles, the more resistant parent. To further investigate this QTL, single-nucleotide polymorphisms (SNPs) located on chromosome 7H were genotyped on the population using competitive allele-specific polymerase chain reaction (KASP) assays. After removal of data for six lines with numerous apparent recombination events on chromosome 7H (probably indicative of DNA or seed contamination), a new linkage map was constructed for chromosome 7H, with 96 SNP markers over 157.4 cM (Table S3). With QTL analysis using this map, the maximum LOD value (48.4) was at 138.9 cM (Figure 2), the same position as marker *wri907*. This QTL was designated *QRlnt.ArFr-7H*.

### 2.3. Genetic Mapping: Denar/Baudin

**Figure 3:**
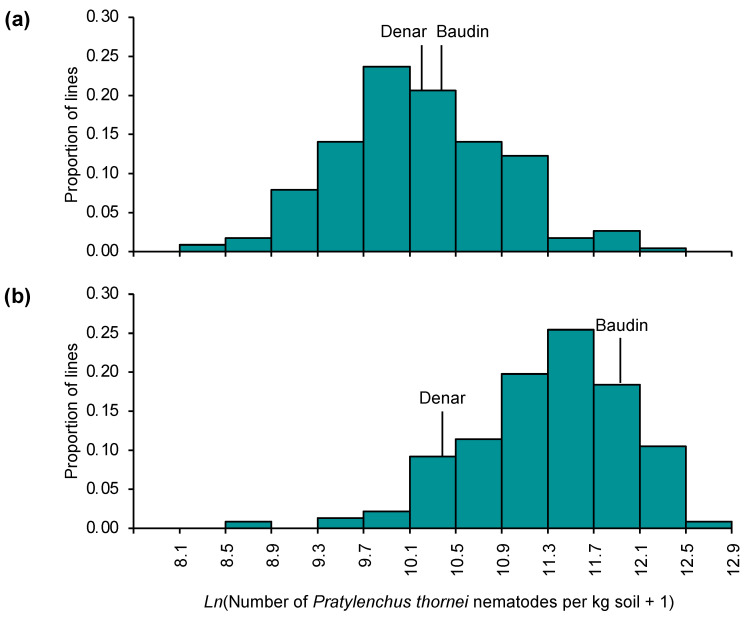
Frequency distributions for estimated final numbers of *Pratylenchus thornei* nematodes at 16 weeks after inoculation for a Denar/Baudin barley population evaluated in two experiments (**a**,**b**).

The Denar/Baudin population was then chosen for QTL mapping because Denar (5.46 *P. thornei* per g of soil and roots) was more resistant than Baudin (13.00 *P. thornei* per g of soil and roots). Denar/Baudin doubled haploid lines were evaluated for *P. thornei* resistance in two 16-week experiments, conducted in successive years in a glasshouse in Queensland. In those experiments, final nematode numbers were determined by counting. In the first experiment, the phenotypic frequency distribution did not deviate significantly from normality (Shapiro–Wilk *W =* 099, *p* = 0.5810) (Figure 3a). In the second experiment, nematode numbers were higher than in the first experiment, and their distribution deviated significantly from normality (Shapiro–Wilk *W =* 0.97, *p* = 0.0001) (Figure 3b).

With QTL analysis using an existing molecular marker map (Table S4) that had been developed to map QTLs for powdery mildew resistance [28] and phenotypic data from the first Denar/Baudin experiment, a major QTL (LOD = 20.6) was mapped at 166.0 cM on chromosome 7H (Figure S2). Two other QTLs were detected, one at 195.1 cM on chromosome 1H (LOD = 3.9) and one at 144.0 cM on chromosome 3H (LOD = 5.9), both with favorable effects from Baudin. Using phenotypic data from the second Denar/Baudin experiment, the only significant QTL was at 115.0 cM on chromosome 3H (LOD = 3.1): 29.0 cM from the QTL detected based on the first experiment. On chromosome 7H, the highest test statistic was at 166.1 cM, but this was not statistically significant (LOD = 1.9).

**Figure 4:**
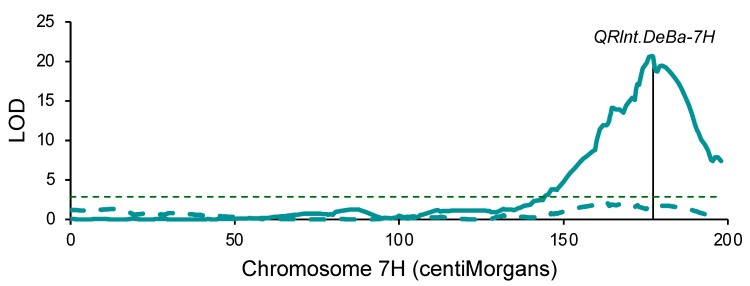
Quantitative trait locus *QRlntDeBa-7H* for *Pratylenchus thornei* resistance in a Denar/Baudin barley population using a linkage map of single-nucleotide polymorphisms and SilicoDArT presence-absence markers on chromosome 7H and phenotypic data from two phenotyping experiments. The solid and dashed scans show LOD test statistic values from the first and second experiments, respectively. The vertical line shows the position of marker *wri907*. The horizontal dashed line shows the genome-wide significance threshold (LOD = 2.87).

With KASP genotyping of SNPs and curation of the existing marker data, the Denar/Baudin map of chromosome 7H was revised to include a total of 120 markers over 197.9 cM (Table S5). Using the revised map, along with phenotypic data from the first Denar/Baudin experiment, a significant QTL (LOD = 20.7) was mapped at 177.0 cM, 0.2 cM proximal to marker *wri907* (Figure 4). This QTL was designated *QRlnt.DeBa-7H*. Using phenotypic data from the second experiment, there was no significant QTL on chromosome 7H (Figure 4).

### 2.4. SNP Genotypes, Physical Positions and Haplotypes

**Figure 5:**
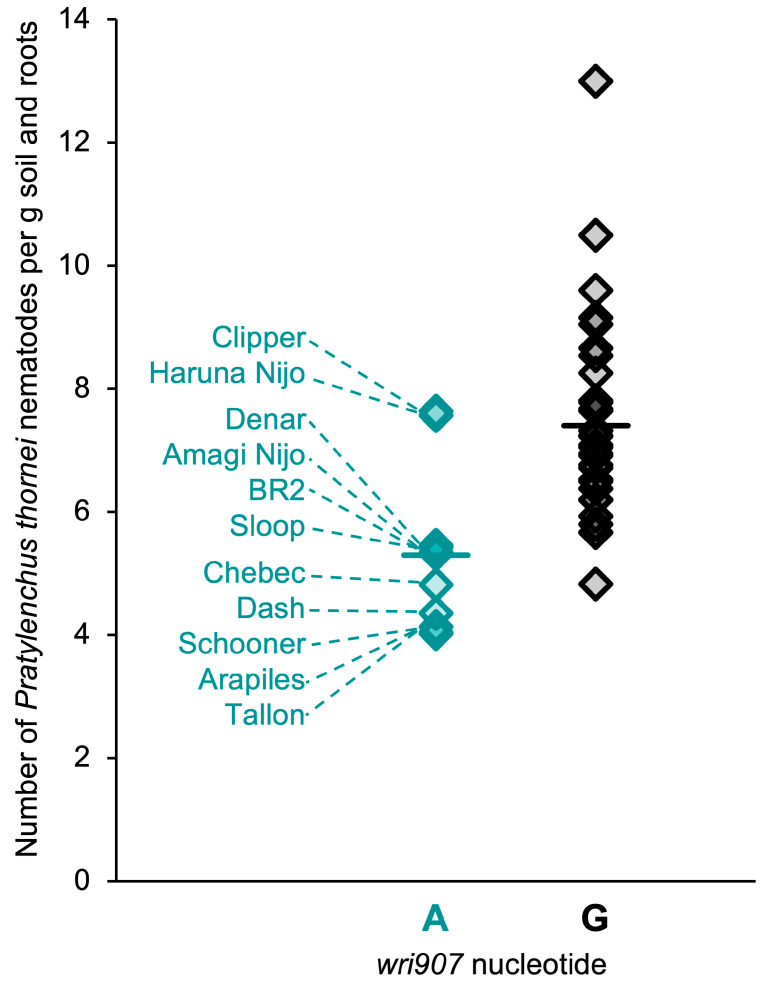
Estimated final numbers of *Pratylenchus thornei* nematodes for 11 barley mapping parents with the resistance-associated nucleotide (A) at marker *wri907* and 35 parents with the alternative nucleotide (G). Solid horizontal lines show the mean values for the two genotypic classes.

Of the 46 mapping parents, 11 (Amagi Nijo, Arapiles, BR2, Chebec, Clipper, Dash, Denar, Schooner, Sloop and Tallon) were found to have the resistance-associated nucleotide (A) at *wri907*. The mean final number of *P. thornei* nematodes was significantly lower for these lines than for lines with the alternative nucleotide (G) (Figure 5).

**Figure 6:**
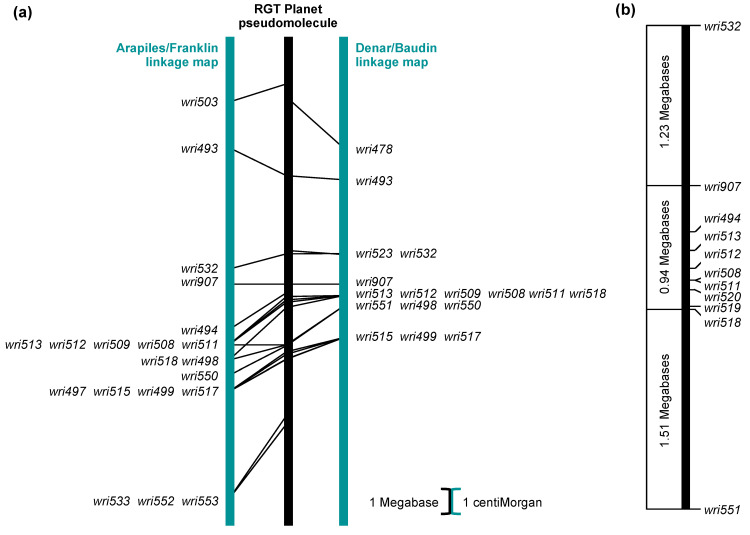
Positions of (**a**) 23 single-nucleotide polymorphisms on two genetic linkage maps and on the 7H pseudomolecule of the RGT Planet genome sequence assembly [23] and (**b**) 11 single-nucleotide polymorphisms in a candidate region of that pseudomolecule.

Among 14 other varieties for which *P. thornei* resistance ratings are available from the Grains Research and Development Corporation [22], 4 (Alestar, Compass, Flinders and RGT Planet) have the resistance-associated nucleotide (A) at *wri907*. All of these have been rated as moderately resistant [22]. One of them (RGT Planet) has been sequenced in a barley pan-genome project [29]. On the RGT Planet 7H pseudomolecule, the *wri907* SNP is at 615,167,850 bp, and 22 other SNPs that were genetically mapped in the QTL region are between 607,064,377 bp (*wri503*) and 620,825,840 bp (*wri553*). The genetic and physical orders of these SNPs are highly collinear (Figure 6a).

**Figure 7:**
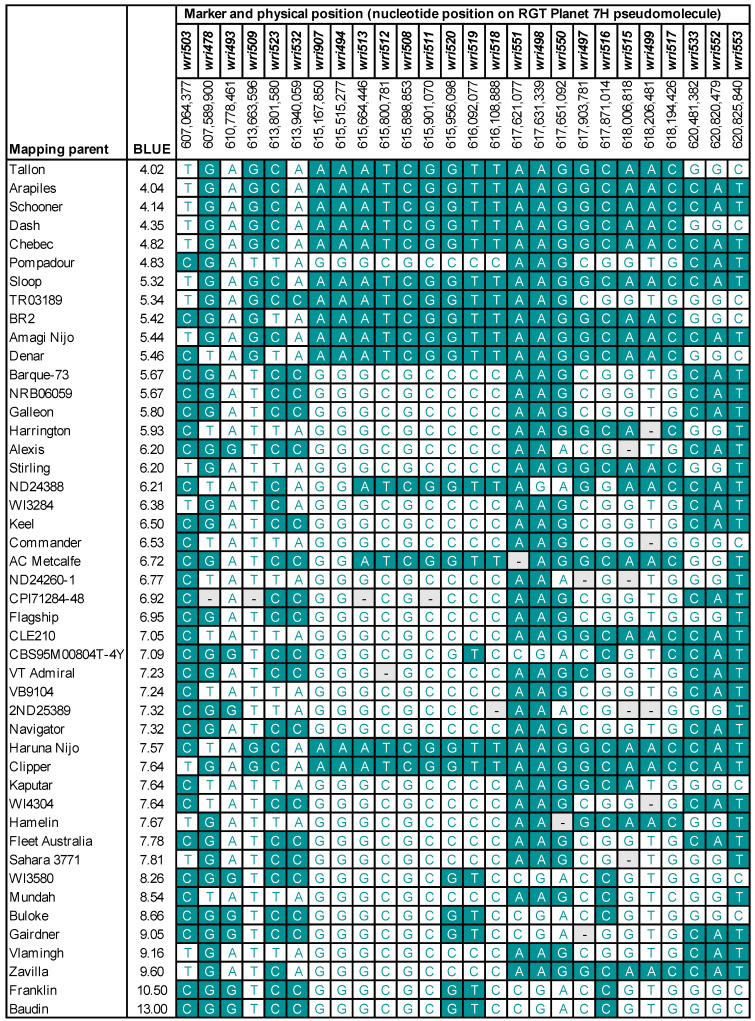
Haplotypes for 46 barley mapping parents across 26 single-nucleotide polymorphism markers. Physical positions are on the RGT Planet 7H pseudomolecule sequence. Parents are listed in order from lowest to highest best linear unbiased estimate (BLUE) of the final number of *Pratylenchus thornei* nematodes, i.e., from most resistant to most susceptible. Nucleotides that are the same as in RGT Planet are shown in white text on a dark background. - = missing genotypic data.

**Figure 8:**
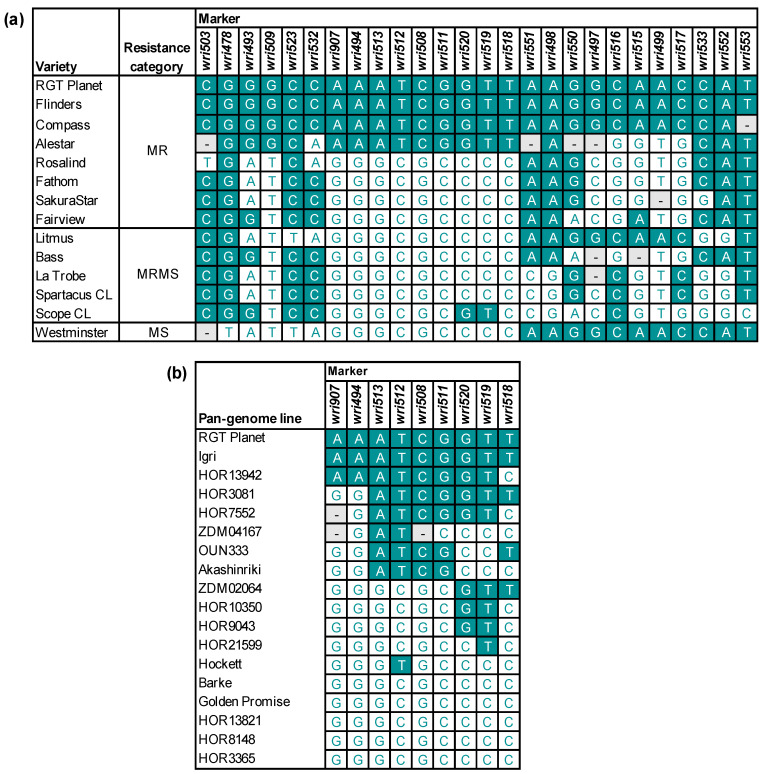
Haplotypes for (**a**) 14 barley varieties across 26 single-nucleotide polymorphism markers, showing the resistance status [22] of each variety as moderately resistant (MR), moderately resistant to moderately susceptible (MRMS) or moderately susceptible (MS) and (**b**) 20 barley pan-genome lines across nine of those markers. All markers are arranged according to their physical order in the RGT Planet 7H pseudomolecule sequence. Nucleotides that are the same as in RGT Planet are shown in white text on a dark background. The resistance status of the pan-genome lines is not known, except for RGT Planet, which is moderately resistant. - = missing genotypic data.

When 26 SNPs were assayed on the 46 mapping parents and 14 barley varieties, nine of the ten most resistant mapping parents and four of eight moderately resistant varieties were found to have identical genotypes for a series of nine SNPs from *wri907* (615,167,850 bp) to *wri518* (616,108,881 bp) (Figure 7 and Figure 8a). For those SNPs, the genotypes of 20 barley pan-genome lines were obtained from genome assemblies. Among these lines, only RGT Planet (moderately resistant to *P. thornei*) and Igri (unknown resistance status) have the resistance-associated haplotype across all nine SNPs (Figure 8b).

Though it seems likely that the causal gene for the QTL is in the 0.94 Mb region between *wri907* and *wri518*, it is possible that it is proximal to *wri907* or distal to *wri518*. To allow for these possibilities, a longer region, including the 0.94 Mb region and its two flanking intervals (Figure 6b), was chosen for further investigation. This region, which spans 3.68 Mb of the RGT Planet 7H pseudomolecule, contains 108 predicted genes (Table S6). Though over half of these have been annotated with descriptions based on sequence similarity, none have proven functions. Of the 108 predicted RGT Planet genes, 56 were matched to predicted genes in the PanBaRT20 linear pan-genome assembly [30]. Though most of these matches were unique, two RGT Planet genes (PLANET_7H01G663800 and PLANET_7H01G664600) were matched with PanBaRT20_7H78697, and two (PLANET_7H01G667900 and PLANET_7H01G668100) were matched with PanBaRT20_7H78730. All but one of the matched PanBaRT20 genes are within a 4.79 Mb region (672.11 to 676.90 Mb) of the PanBaRT20 assembly. The exception is PanBaRT20_7H77511 at 642.40 Mb, which was matched with PLANET_7H01G664900. Examination of barley pan-transcriptomic data [30] revealed that 34 of the 54 matched PanBaRT20 genes expressed at least one transcript per million (TPM) in RGT Planet root tissue (Table 1). For 15 of these genes, expression was significantly higher in RGT Planet than in at least 1 of the other 19 lines (Table 2). For 11 genes, expression was significantly lower in RGT Planet than in at least 1 of the other 19 lines (Table 3). 

## 3. Discussion

### 3.1. Mapping of a Common Quantitative Trait Locus in Two Barley Populations

Given a lack of prior genetic information on *P. thornei* resistance in barley, this research started with phenotypic evaluation of a panel of barley lines. To make it possible to proceed to QTL mapping without having to develop new experimental material, the lines chosen for this panel had all previously been used as parents to develop mapping populations. Well-characterized wheat varieties were included as controls, making it possible to see that the range of *P. thornei* resistance in barley is similar to that in wheat. Based on the panel results, two cross combinations were chosen for further analysis: Arapiles/Franklin and Denar/Baudin. Populations derived from each of these had previously been genotyped for molecular markers, providing genetic linkage maps that have been used to map QTLs for other traits [27,28].

### 3.2. Mapping of a Common Quantitative Trait Locus in Two Barley Populations

For the Arapiles/Franklin population, genotyping had been performed with older marker types (amplified fragment length polymorphisms (AFLPs) and simple sequence repeats (SSRs)), and the linkage map was quite sparse. Nevertheless, a very significant QTL for *P. thornei* resistance was detected on chromosome 7H. Genotyping of chromosome 7H SNPs using KASP assays enabled the development of an improved linkage map of chromosome 7H. In contrast to the original map, which has 58 AFLP markers and two SSR markers over 245.1 cM and eight intervals longer than 10 cM, the SNP-based map has 166 markers over 154.7 cM and just two intervals longer than 10 cM. The peak QTL test statistic is higher on the new map (LOD = 48.4 compared to 36.3), perhaps because the SNP genotyping was more accurate and complete than the AFLP and SSR genotyping. 

For the Denar/Baudin population, the genetic map of chromosome 7H provided good resolution (126 DArTseq markers over 189.8 cM) for QTL mapping. Revising this map to exclude redundant DArTseq markers and to include SNPs genotyped with KASP assays had little effect on QTL test statistic values but facilitated comparison with the revised Arapiles/Franklin map.

For both *QRlnt.ArFr-7H* and *QRlnt.DeBa-7H* (the *P. thornei* resistance QTLs mapped on the long arm of chromosome 7H in the Arapiles/Franklin and Denar/Baudin populations, respectively), w*ri907* is the closest marker to the estimated QTL positions. This collocation, in combination with Arapiles and Denar having identical genotypes at 21 consecutive SNP markers in the QTL region, indicates that the same resistance gene is segregating in the two populations. This was not expected, given that the Australian variety Arapiles (pedigree Noyep/Proctor//CI3576/Union/4/Kenia/3/Research/2/Noyep/Proctor/5/Domen) and the Czech variety Denar (pedigree Celechovicky Hanacky/Bavaria) are not known to be related.

The exact collocation of *QRlnt.ArFr-7H* and *QRlnt.DeBa-7H* occurred despite different methods having been used to phenotype the two populations. This similarity of results is consistent with a previous report that the standard methods used in southern and northern Australia provide highly correlated results [25,26]. Surprisingly, though, *QRlnt.DeBa-7H* was statistically significant in only the first of two Denar/Baudin experiments, despite the experiments having been conducted in the same manner. One possible explanation for this could be that environmental conditions in the second year were more favorable for *P. thornei*, allowing it to largely overcome the resistance conferred by *QRlnt.DeBa-7H.* Consistent with this interpretation, the final nematode numbers tended to be higher in the second experiment than in the first experiment. 

### 3.3. A Candidate Region and Predicted Genes on a Barley Genome Assembly

RGT Planet, which has been rated as having moderate *P. thornei* resistance [18] and has a similar haplotype to Arapiles and Denar in the QTL region, may carry the same resistance gene as Arapiles and Denar. RGT Planet was among the materials used in a recent pan-genome sequencing effort [23] that provided genome assemblies for 20 barley lines. With anchoring of QTL-linked SNPs to the RGT Planet 7H pseudomolecule, a 3.68 Mb candidate region was defined for *QRlnt.ArFr-7H* and *QRlnt.DeBa-7H*. Within this region, the RGT Planet sequence and the Arapiles/Franklin and Denar/Baudin genetic maps are highly collinear.

Evaluation of the predicted genes within the candidate region provided a list of potential candidate genes. With none of those genes having proven functions, many of those genes not having been annotated with a description, no previously discovered genes for root-lesion nematode resistance in any host plant species and little known about *P. thornei* resistance mechanisms in barley, there is little basis on which to narrow the list based on gene function. Two genes in the region have descriptions that could indicate a role in plant defense (PLANET_7H1G01G662000 (receptor-like kinase) and PLANET_7H01G668300 (leucine-rich repeat receptor-like protein kinase family protein, putative). It is not known whether resistance against root-lesion nematodes operates in a similar manner to resistance against fungal and bacterial pathogens, but receptor-like kinases have previously been suggested as candidate genes for *P. thornei* resistance in wheat [18] and chickpea [31] and have been reported to be overexpressed in chickpea after infection by *P. thornei* [32]. In wheat, it has been shown that resistance acts after the nematodes penetrate roots, indicating that resistance could involve constitutively expressed water-soluble compounds that inhibit egg hatching and the development of juvenile nematodes [33]. Consistent with this, genes encoding enzymes in the biosynthesis of flavonoids and isoflavonoids (isoflavone reductase, flavonoid 3′-hydroxylase, chalcone synthase and phenylalanine ammonia-lyase) have been discussed as candidates for *P. thornei* resistance QTLs in wheat [18]. However, none of the predicted genes in the RGT Planet candidate region have been annotated as encoding such enzymes.

Mining of existing barley pan-transcriptomic data [30] provided some information on gene expression in root tissue of RGT Planet and 19 other barley lines. Unfortunately, interpretation of expression levels and differential expression is limited by several factors. Firstly, it cannot be assumed that the causal gene is highly expressed in root tissue or that it is differentially expressed between lines that differ in resistance. Secondly, the analysis approach used here could not provide expression data for RGT Planet genes that could not be matched with PanBaRT20 genes. If those genes are truly present in RGT Planet but not in many other barley lines, they could be good candidates for the resistance gene. Thirdly, interpretation of differential expression results is complicated by the fact that RGT Planet is the only one of the 20 pan-genome lines for which there is information on *P. thornei* resistance. Despite these limitations, genes for which expression in RGT Planet is higher or lower than other lines could be interesting candidates. One such gene is PLANET_7H01G661600, which was annotated as a putative kinase. For that gene, expression was significantly higher in RGT Planet than in 16 of the other 19 lines: all except Igri (which has the same nine-SNP haplotype as RGT Planet), OUN333 and the landrace HOR7552. Other potentially interesting genes in the same interval include PLANET_7H01G660900 and PLANET_7H01G661100 (both with significantly higher expression in RGT Planet than in 11 other lines) and PLANET_7H01G658200 (lysosomal Pro-X carboxypeptidase), for which expression in RGT Planet was significantly lower than that in nine other lines. 

Based on the results reported here, it would be unrealistic to propose a short list of candidate genes, but the information presented in Table 1, Table 2, Table 3 and Table S6 could support the formulation of hypotheses for future experimentation. In future research, Igri and other pan-genome lines could be evaluated for resistance to *P. thornei*. In addition, transcriptomic analysis could be conducted for root tissue sampled from resistant and susceptible materials grown with and without *P. thornei*. It would be particularly interesting to know whether expression of the putative receptor-like kinase (PLANET_7H01G66200) or the putative leucine-rich repeat receptor-like protein kinase (PLANET_7H01G668300) is up-regulated in response to infection. Alternatively, or in parallel, attempts could be made to genetically narrow the candidate region. This would require generation of new progeny, which could be genotyped to identify new recombinants. Homozygous recombinants could then be phenotyped. Fine mapping of the resistance locus would be facilitated if the trait could be “Mendelised”, allowing for classification of progeny into two resistance categories. Considering the bimodal phenotypic distribution observed for the Arapiles/Franklin population, it might be possible to achieve this, perhaps by increasing replication in the phenotyping protocol. 

### 3.4. Application in Barley Breeding

In breeding materials segregating for the *QRlnt.ArFr-7H*/*QRlnt.DeBa-7H* resistance locus, a marker assay such as *wri907* could be used to select progeny with the resistance allele. In Australia, *P. thornei* resistance currently does not rank highly as a breeding objective for barley. Phenotyping for resistance is expensive, and new grain crop varieties are typically not evaluated for root-lesion nematode resistance until just before, or even after, they are released for production. Marker-assisted selection in early generations could provide a cost-effective way to improve or maintain *P. thornei* resistance in barley. This could help limit losses in barley crops. Further, by reducing *P. thornei* populations in the soil, the use of resistant barley varieties could benefit subsequent crops in agricultural rotations.

### 3.5. Alternative Sources of Resistance

Though the results presented here provide strong evidence that an Arapiles/Denar/RGT Planet haplotype in the *QRlnt.ArFr-7H*/*QRlnt.DeBa-7H* region is associated with *P. thornei* resistance, they also show that some moderately resistant barley varieties do not carry this haplotype. This seems to indicate that the *QRlnt.ArFr-7H*/*QRlnt.DeBa-7H* region is not the only one conferring *P. thornei* resistance in barley. Among the materials evaluated here, possible sources of resistance that cannot be attributed to the *QRlnt.ArFr-7H*/*QRlnt.DeBa-7H* QTL include the Australian varieties Fairview, Fathom, Rosalind and SakuraStar. 

## 4. Materials and Methods

### 4.1. Plant Materials

The plant materials used in this research included the following:

(1) A panel of 46 barley lines (Table S1), each of which had previously been used as a parent to develop one or more mapping populations;
(2) Eight varieties of bread wheat: Catalina, Chara, EGA Gregory, Estoc, Mace, Machete, Estoc, Naparoo and Yandanooka (Table S1);
(3) One variety of durum wheat (*T. turgidum* ssp. *durum* L.): Tamaroi (Table S1);
(4) 225 doubled haploid lines derived from the F_1_ generation of a cross between Arapiles and Franklin (Table S2);
(5) 235 doubled haploid lines derived from the F_1_ generation of a cross between Denar and Baudin (Table S4);
(6) 14 other barley varieties for which *P. thornei* resistance ratings are available from the Australian Grains Research and Development Corporation [22] (Figure 8).

### 4.2. Evaluation of Pratylenchus thornei Resistance in South Australia

The 46 barley mapping parents and nine wheat varieties (Table S1) and, subsequently, Arapiles, Franklin and 169 Arapiles/Franklin doubled haploid lines were evaluated for *P. thornei* resistance using methods similar to those that were previously described [25,26] as being used in South Australia, except that DNA was extracted from soil (including roots) rather than washing soil from the roots and extracting DNA from the roots. Square plastic pots (55 mm wide by 120 mm high) were filled with pasteurized sand. The pots were arranged in purpose-built galvanized steel square (5 × 5) mesh crates and placed in a greenhouse maintained at 20° ± 3 °C. One pre-germinated seed was sown per pot, with lines assigned to pots according to randomized complete block designs, with six complete blocks for the mapping parents and wheat varieties and four complete blocks for the Arapiles/Franklin population.

After seedlings emerged, inoculum was pipetted into two 5 cm deep holes on either side of each seedling, with approximately 1500 nematodes applied per plant. The isolate used (Pt9EP) was originally collected on the Eyre Peninsula, South Australia and had been maintained on carrot callus cultures [34]. One week after inoculation, slow-release fertilizer was added and covered with 1 cm of sand. Crates were placed in ebb-flow trays that were flooded for 4 min every 3 days to a depth of 10 cm using water from a reservoir below each tray. 

At eight weeks after inoculation, shoots were removed, and soil (including roots) was removed from each pot and dried at 48 °C. Dried samples were provided to the South Australian Research and Development Institute Molecular Diagnostic Center (https://pir.sa.gov.au/research/services/molecular_diagnostics (accessed on 25 January 2024)) for DNA extraction and application of a Predicta assay [25] to quantify *P. thornei* DNA. For each of the mapping parents, the Predicta assay was applied to eight aliquots of DNA: two from each of two blocks and one from each of the other four blocks. For each doubled haploid line, the Predicta assay was applied to one DNA aliquot per pot. Measurements were converted to nematode equivalents (per g of soil and roots) using standard curves that had been developed using soil samples to which known numbers of nematodes had been added [25]. BLUEs were calculated for numbers of *P. thornei* nematodes per g of soil and roots.

### 4.3. Evaluation of Pratylenchus thornei Resistance in Queensland

Denar, Baudin and the Denar/Baudin doubled haploid lines were evaluated using methods that have previously been described [26] as being used in the Queensland. Two experiments were grown in successive years, each with entries arranged in three randomized complete blocks. Plants were grown in square pots (70 mm wide by 150 mm high), each containing 330 g (oven-dry equivalent) pasteurized vertosolic soil mixed with slow-release fertilizer. Pots containing 80% of the final amount of soil were arranged on greenhouse benches fitted with a bottom-watering system regulated by a float valve set to a water tension of 2 cm. Three seeds of the same line were placed onto the soil surface in each pot.

Inoculum containing approximately 3300 nematodes was pipetted around the seeds. The inoculum originated from 10 specimens collected near Jondaryn, Queensland. After inoculation, the remaining soil was placed over the seed. After emergence, plants were removed as needed to leave one plant per pot, by cutting below the seed (leaving the roots). Soil temperature was maintained at 22 °C by under-bench heating. Maximum air temperature was maintained between 20 and 25 °C by using shade cloth (as required) and evaporative coolers. 

After 16 weeks of plant growth, the soil and roots from each pot were thoroughly mixed, and the roots cut into lengths of about 1 cm. Nematodes were extracted from a 150 g subsample at 22 °C for 48 h using the Whitehead tray method [35], and nematodes were collected on a 20 μm sieve. Samples were stored in 30 mL vials at 3 °C. Nematodes extracted from soil and roots were counted once using a 1 mL Hawksley-type nematode counting chamber (Chalex Corporation, Wallowa, OR, USA) under a compound microscope. BLUEs were calculated for *ln*(*x* + 1) transformations [36] of numbers of *P. thornei* nematodes per kg of soil.

### 4.4. Genetic Mapping, Marker Development and Genotyping

Initial QTL analysis was conducted using existing genotypic data and linkage maps. The Arapiles/Franklin linkage map (Table S2) consisted of SSR markers and AFLP markers [27]. The Denar/Baudin linkage map (Table S4) consisted of SNP and SilicoDArT markers [28].

Quantitative trait locus analysis was conducted by simple interval mapping using the R/qtl package [37] in the R Statistical Computing Environment [38]. Significance of LOD test statistic values was evaluated relative to thresholds obtained using 10,000 permutations and a genome-wide significance level of 0.05. 

Following detection of a common QTL on chromosome 7H, SNPs from the QTL region were genotyped on one or both mapping populations, depending on whether they were polymorphic between the parents. In addition, SNPs from other regions of chromosome 7H were genotyped on the Arapiles/Franklin population. The SNPs were genotyped with KASP assays [39] using an automated SNPline system (LGC Biosearch Technologies, Teddington, UK) according to the manufacturer’s instructions. Primer sequences are given in Table S7.

For the Arapiles/Franklin population, a new linkage map was constructed for chromosome 7H using data for SNP markers that had been genotyped with KASP assays. For the Denar/Baudin population, the linkage map of chromosome 7H was revised, using data for SNP markers that had been genotyped with KASP assays in combination with data for DArTseq SNP markers (excluding any for which a KASP assay had been developed and applied) and for DArTseq SilicoDArT markers. Marker loci were ordered, and genetic distances calculated using the MSTmap algorithm [40] as implemented in the R/ASMap package [41]. The resulting maps were examined, and some markers were removed from the datasets. For the Denar/Baudin map, SilicoDArT markers were retained only if they mapped in regions with no SNP markers. For both maps, positions with more than one mapped marker were identified. If the position was in the QTL region, all markers were retained. Elsewhere on the chromosome, only the marker with the most complete data was retained. The remaining data were then re-analyzed to finalize marker orders and map positions.

### 4.5. Analysis of Pan-Genomic and Pan-Transcriptomic Data

To determine the physical positions of SNPs in a QTL region on the long arm of chromosome 7H, SNP-bearing sequences were BLASTed [42] against version 1 of the RGT Planet barley genome assembly [23] using SequenceServer [43] at GrainGenes [44]. Once a candidate region was defined on the RGT Planet 7H pseudomolecule, information on predicted genes in that region was downloaded from GrainGenes. The sequences of those genes were mapped to a PanBaRT20 linear pan-genome assembly [30] using Minimap2 software [45]. Each RGT Planet gene that overlapped with a PanBaRT20 gene was considered to match that PanBaRT20 gene if the overlap length exceeded 30% of the length of each gene. This filtering threshold corresponds with the 25th percentile of the overlap length distribution.

For each matched gene, gene expression data were extracted from a set of pan-transcriptomic data for RGT Planet and 19 other barley lines. Differential expression analysis was conducted using 3D RNA-seq software [46]. Nineteen contrasts were defined (e.g., Barke vs. RGT Planet, Morex vs. RGT Planet), enabling comparisons of expression in RGT Planet to expression in each of the other 19 lines. For each comparison, a gene was considered to be differentially expressed if its Benjamini–Hochberg-adjusted *p* value was below 0.05 and the absolute value of the log_2_ fold-change was greater than or equal to 1.

## Figures and Tables

**Table 1:** Genes between 613,940,059 bp and 617,621,077 bp on the RGT Planet 7H pseudomolecule sequence [23] for which the corresponding gene in the PanBaRT20 barley pan-transcriptome assembly [24] expressed an average of at least 1 transcript per million (TPM) in RGT Planet root tissue.

RGT Planet Gene		PanBaRT20 Gene ID	Mean TPM
Gene ID	Description		
PLANET_7H01G664900	Carbonic anhydrase	PanBaRT20_7H77511	120.52
PLANET_7H01G657100	WRKY transcription factor	PanBaRT20_7H78604	79.51
PLANET_7H01G657200	WRKY transcription factor	PanBaRT20_7H78606	171.32
PLANET_7H01G657500		PanBaRT20_7H78609	6.04
PLANET_7H01G658100	Aldehyde oxidase, putative	PanBaRT20_7H78620	5.59
PLANET_7H01G658300	Lysosomal Pro-X carboxypeptidase	PanBaRT20_7H78624	2.79
PLANET_7H01G658600	50S ribosomal protein L35	PanBaRT20_7H78626	6.39
PLANET_7H01G659700	50S ribosomal protein L35	PanBaRT20_7H78646	4.49
PLANET_7H01G660900		PanBaRT20_7H78652	32.41
PLANET_7H01G661100		PanBaRT20_7H78658	60.94
PLANET_7H01G661600	Kinase, putative	PanBaRT20_7H78661	4.19
PLANET_7H01G661800	Homeobox protein knotted-1, putative	PanBaRT20_7H78662	102.55
PLANET_7H01G661900	Kinase family protein	PanBaRT20_7H78663	16.81
PLANET_7H01G662100	Indole-3-glycerol phosphate synthase-like	PanBaRT20_7H78670	72.89
PLANET_7H01G662400	NHL domain-containing protein, putative	PanBaRT20_7H78676	332.46
PLANET_7H01G664400		PanBaRT20_7H78691	2.96
PLANET_7H01G664500		PanBaRT20_7H78695	8.51
PLANET_7H01G665100	Methyltransferase-like	PanBaRT20_7H78700	4.91
PLANET_7H01G665200	Ribonuclease 3-like protein 3	PanBaRT20_7H78702	18.89
PLANET_7H01G666100	Methyl-CpG-binding domain protein	PanBaRT20_7H78705	22.02
PLANET_7H01G666300	Transcription initiation factor TFIID subunit 1	PanBaRT20_7H78707	19.99
PLANET_7H01G666400	XH/XS domain-containing family protein	PanBaRT20_7H78709	28.05
PLANET_7H01G666500	Transcription initiation factor TFIID subunit 1	PanBaRT20_7H78710	21.36
PLANET_7H01G666600	Transmembrane protein	PanBaRT20_7H78711	23.57
PLANET_7H01G666900		PanBaRT20_7H78714	1.82
PLANET_7H01G667300	NF-X1-type zinc finger protein NFXL1	PanBaRT20_7H78717	3.95
PLANET_7H01G667400	Isoleucine–tRNA ligase	PanBaRT20_7H78719	70.82
PLANET_7H01G667500	Mis12 protein	PanBaRT20_7H78720	26.22
PLANET_7H01G667900, PLANET_7H01G668100		PanBaRT20_7H78730	353.53
PLANET_7H01G668200		PanBaRT20_7H78735	42.43
PLANET_7H01G668400	Amino acid transporter-like protein	PanBaRT20_7H78741	23.33
PLANET_7H01G668500	WD40 repeat-like protein	PanBaRT20_7H78742	31.70
PLANET_7H01G668800	S-acyltransferase	PanBaRT20_7H78743	26.43
PLANET_7H01G668900	Pyrophosphate-energized vacuolar	PanBaRT20_7H78744	183.49

**Table 2:** Genes between 613,940,059 bp and 617,621,077 bp on the RGT Planet 7H pseudomolecule sequence [23] for which the corresponding gene in the PanBaRT20 barley pan-transcriptome assembly [24] expressed at least 1 transcript per million in RGT Planet root tissue and had significantly higher expression in RGT Planet than in at least 1 of 19 other barley pan-transcriptome lines.

RGT Planet Gene		PanBaRT20 Gene ID	Number of Lines with Lower Expression than RGT Planet ^1^
Gene ID	Description		
PLANET_7H01G664900	Carbonic anhydrase	PanBaRT20_7H77511	4
PLANET_7H01G657100	WRKY transcription factor	PanBaRT20_7H78604	9
PLANET_7H01G657200	WRKY transcription factor	PanBaRT20_7H78606	7
PLANET_7H01G659700	Kinase family protein	PanBaRT20_7H78646	1
PLANET_7H01G660900		PanBaRT20_7H78652	11
PLANET_7H01G661100		PanBaRT20_7H78658	11
PLANET_7H01G661600	Kinase, putative	PanBaRT20_7H78661	16
PLANET_7H01G661800	Homeobox protein knotted-1, putative	PanBaRT20_7H78662	1
PLANET_7H01G662100	Indole-3-glycerol phosphate synthase-like	PanBaRT20_7H78670	2
PLANET_7H01G662400	NHL domain-containing protein, putative	PanBaRT20_7H78676	7
PLANET_7H01G664400		PanBaRT20_7H78691	2
PLANET_7H01G664500		PanBaRT20_7H78695	7
PLANET_7H01G666600	Transmembrane protein	PanBaRT20_7H78711	7
PLANET_7H01G667900 PLANET_7H01G668100		PanBaRT20_7H78730	14
PLANET_7H01G668900	Pyrophosphate-energized vacuolar membrane proton pump	PanBaRT20_7H78744	2

^1^ Lines for which the Benjamini–Hochberg-adjusted *p* value was below 0.05 and the absolute value of the log_2_ fold-change was greater than or equal to 1.

**Table 3:** Genes between 613,940,059 bp and 617,621,077 on the RGT Planet 7H pseudomolecule sequence [23] for which the corresponding gene in the PanBaRT20 barley pan-transcriptome assembly [24] had significantly lower expression in root tissue of RGT Planet than in root tissue of least 1 of 19 other barley pan-transcriptome lines.

RGT Planet Gene		PanBaRT20 Gene ID	Number of Lines with Higher Expression than RGT Planet ^1^	Absolute Value of log_2_ Fold-Change	
Gene ID	Description			Maximum	Median ^2^
PLANET_7H01G664900	Carbonic anhydrase	PanBaRT20_7H77511	1	1.56	1.56
PLANET_7H01G657100	WRKY transcription factor	PanBaRT20_7H78604	2	1.23	1.21
PLANET_7H01G657500		PanBaRT20_7H78609	4	1.16	1.04
PLANET_7H01G658100	Aldehyde oxidase, putative	PanBaRT20_7H78620	8	2.90	1.67
PLANET_7H01G658200	Lysosomal Pro-X carboxypeptidase	PanBaRT20_7H78622	9	3.66	1.93
PLANET_7H01G658300	Lysosomal Pro-X carboxypeptidase	PanBaRT20_7H78624	1	1.34	1.34
PLANET_7H01G660900		PanBaRT20_7H78652	1	1.25	1.25
PLANET_7H01G662100	Indole-3-glycerol phosphate synthase-like	PanBaRT20_7H78670	2	1.37	1.24
PLANET_7H01G662400	NHL domain-containing protein, putative	PanBaRT20_7H78676	1	1.20	1.20
PLANET_7H01G664400		PanBaRT20_7H78691	1	1.07	1.07
PLANET_7H01G663800 PLANET_7H01G664600	Glycosyltransferase	PanBaRT20_7H78697	5	1.82	1.54
PLANET_7H01G666900		PanBaRT20_7H78714	1	1.02	1.02

^1^ Lines for which the Benjamini–Hochberg-adjusted *p* value was below 0.05 and the absolute value of the log_2_ fold-change was greater than or equal to 1. ^2^ Median of values greater than or equal to 1.

## Data Availability

The original contributions presented in the study are included in the article/Supplementary Materials. Further inquiries can be directed to the corresponding author.

## Supplementary Materials

The following supporting information can be downloaded at https://www.mdpi.com/article/10.3390/plants13121663/s1, Figure S1: Chromosome 7H LOD test statistic scan for *Pratylenchus thornei* resistance in an Arapiles/Franklin barley mapping population. Genetic positions shown on the horizontal axis are for a linkage map of amplified fragment length polymorphism and simple sequence repeat markers; Figure S2: LOD test statistic scans for barley chromosomes on which significant quantitative trait loci were detected for a Denar/Baudin barley mapping population based on phenotypic data from two experiments and using a previously published linkage map; Table S1: Barley mapping parents and wheat varieties evaluated for *Pratylenchus thornei* resistance; Table S2: Phenotypic data, genotypic data and a previously published genetic linkage map for an Arapiles/Franklin barley mapping population; Table S3: Phenotypic data for *Pratylenchus thornei* resistance, genotypic data for single-nucleotide polymorphisms (SNPs) and a genetic linkage map of SNPs on chromosome 7H for an Arapiles/Franklin barley mapping population; Table S4: Phenotypic data for *Pratylenchus thornei* resistance, genotypic data and a previously published genetic linkage map for a Denar/Baudin barley mapping population; Table S5: Phenotypic data for *Pratylenchus thornei* resistance, genotypic data and a genetic linkage map of chromosome 7H for a Denar/Baudin barley mapping population; Table S6: Predicted genes within a 3.68 Mb candidate region of the RGT Planet 7H pseudomolecule sequence, showing the PanBaRT20 genes with which they were matched and information on the expression of those PanBaRT20 genes in root tissue of RGT Planet and 19 other barley pan-genome lines; Table S7: Markers assayed using competitive allele-specific polymerase chain reaction (KASP) technology.
