# Supplementary material for: A Quantitative Trait Locus with a Major Effect on Root-Lesion Nematode Resistance in Barley
Source: Plants (Basel). 2024 Jun 15;13(12):1663. doi: 10.3390/plants13121663 (PMC11207570; doi:10.3390/plants13121663)
Supplement: Supplementary file 1 [file plants-13-01663-s001.zip › REVISED Figure S1.pdf]

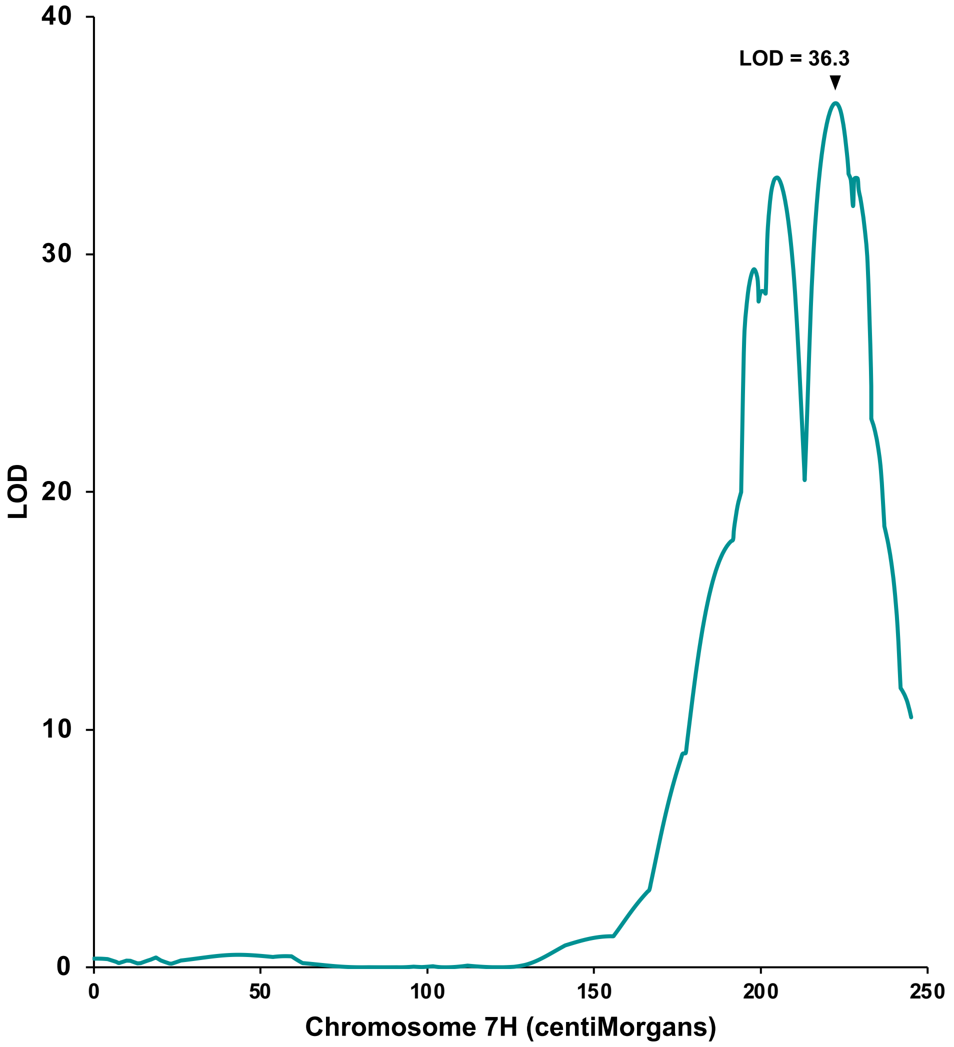

Figure S1. Chromosome 7H LOD test statistic scan for *Pratylenchus thornei* resistance in an Arapiles/Franklin barley mapping population. Genetic positions shown on the horizontal axis are for a linkage map of amplified fragment length polymorphism and simple sequence repeat markers.
