# Supplementary material for: A Quantitative Trait Locus with a Major Effect on Root-Lesion Nematode Resistance in Barley
Source: Plants (Basel). 2024 Jun 15;13(12):1663. doi: 10.3390/plants13121663 (PMC11207570; doi:10.3390/plants13121663)
Supplement: Supplementary file 1 [file plants-13-01663-s001.zip › REVISED Figure S2.pdf]

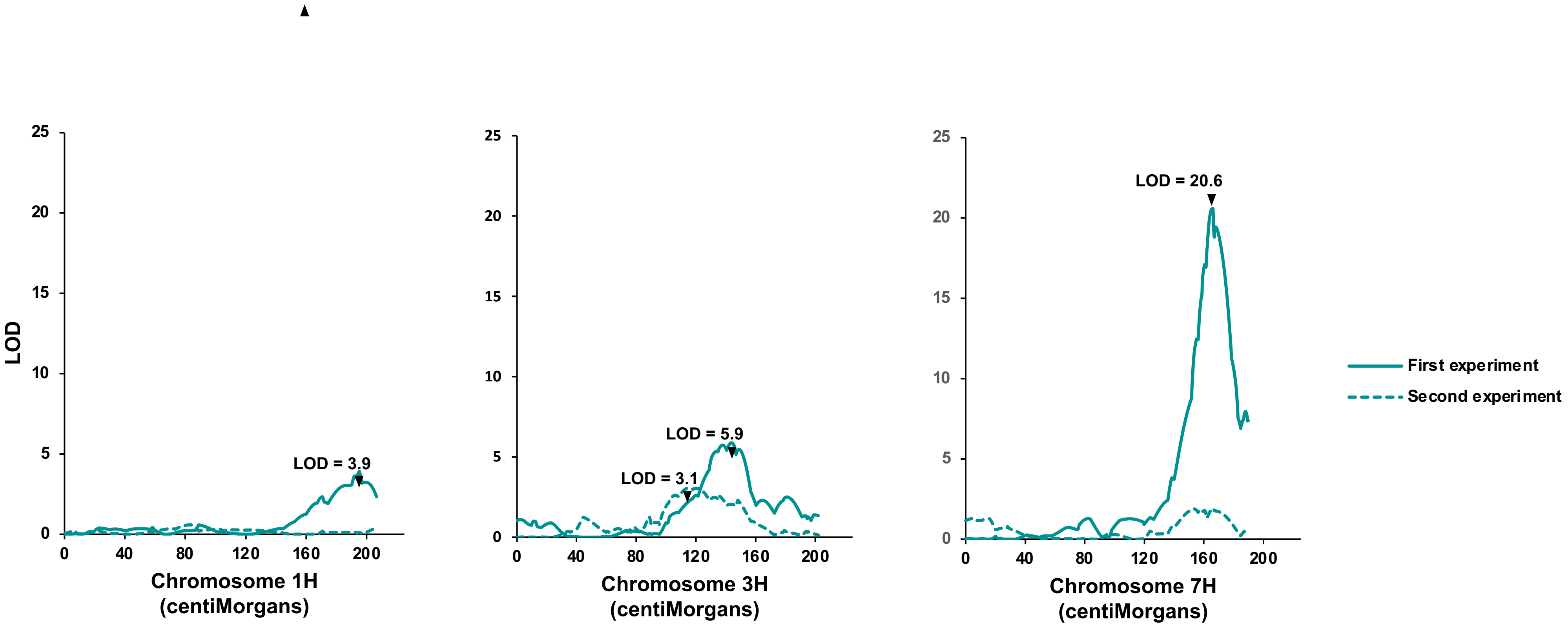

Figure S2. LOD test statistic scans for barley chromosomes on which significant quantitative trait loci were detected for a Denar/Baudin barley mapping population based on phenotypic data from two experiments and using a previously published linkage map.
